# 

**Published:** 1816-01

**Authors:** 


### LONDON MEDICAL AND PHYSICAL JOURNAL;

CONTAINING

ORIGINAL CORRESPONDENCE of EMINENT PRACTITIONERS,

AND THE

EARLIEST INFORMATION ON SUBJECTS CONNECTED WITH

MEDICINE, SURGERY, CHEMISTRY, PHARMACY, BOTANY, AND NATURAL HISTORY.

*Successively conducted by*

T. BRADLEY, M.D.

A. F. M. WILLICH, M.D.

R. BATTY, M.D.

A. A. NOEHDEN, M.D.

J. ARNEMAN, M.D.

J. P. PFAFF, M.D.

J. ADAMS, M.D.

W. SHEARMAN, M.D.

W. ROYSTON, Esq.

AND

SAMUEL FOTHERGILL, M.D.

Assisted by eminent Gentlemen, in the various Branches of the Profession.

VOL. XXXV.

FROM JANUARY TO JUNE, 1816.

5141

Et quoniam variant morbi, variabimus artes;

Mille mali species, mille salutis erunt.

INDEXED

*LONDON:*

PRINTED FOR THE PROPRIETORS, BY J. ADLARD, 23, BARTHOLOMEW-CLOSE, AND 39, DUKE-STREET, SMITHFIELD.

PUBLISHED BY J. SOUTER, NO. 1, PATERNOSTER-ROW; AND MAY BE HAD OF ALL BOOKSELLERS.

W1
20 31

[Entered at Stationers Hall.]

### MONTHLY CATALOGUE OF MEDICAL BOOKS.

A Conspectus of the Pharmocopœias of the London, Edinburgh, and Dublin Colleges of Physicians; being a Practical Compendium of Materia Medica and Pharmacy. By Anthony Todd Thomson, F.L.S. &c. &c. Second edition, corrected and greatly improved. 18mo.—Underwood.

A System of Human Anatomy. By John Gordon, M.D. F.R.S.E. &c. &c. Vol. I. 8vo.—Cadell and Davies.

First Lines of the Practice of Physic, by William Cullen, M.D. including the Definations of the Nosology; with an Appendix, chiefly selected from recent Authors who have contributed to the improvement of Medicine. By Peter Reid, M.D. A new edition, in 2 vols. 8vo.—Longman and Co.

An Account of Two successful Operations for restoring a lost Nose from the Integuments of the Forehead, in the Cases of two Officers of his Majesty's Army. By J. C. Carpue. With Engravings, by Turner, illustrating the different Stages of the Cure.

A Catalogue of a Modern Collection of Books in Anatomy, Medicine, Surgery, Chemistry, Botany, Veterinary Art, &c. containing the most approved Authors; to which is added, a complete List of the Lectures delivered in London. By J. Callow, Medical Bookseller, No. 10, Crown-court, Princes'-street, Soho.

---

### *NOTICES TO CORRESPONDENTS.*

"A Subscriber" *cannot expect us to enter into the complaints of every apprentice. If he is bound to a freeman of London, he must apply to the Chamberlain; if by private indentures, to a justice of the peace.*

*Want of room obliges us to postpone a Report we have received from the Small-Pox Hospital, and also the yearly Bill of Mortality, with our remarks.*

*The papers we have received from the West require our very serious consideration. We are to acknowledge a paper from* Dr. Adams, *from* J. Barlow, Esq., J. Robertson, Esq., *and several others, which will appear in our next.*

